# Genome-Wide Association Study Reveals Key Genes for Differential Lead Accumulation and Tolerance in Natural *Arabidopsis thaliana* Accessions

**DOI:** 10.3389/fpls.2021.689316

**Published:** 2021-08-06

**Authors:** Sílvia Busoms, Laura Pérez-Martín, Miquel Llimós, Charlotte Poschenrieder, Soledad Martos

**Affiliations:** ^1^Plant Physiology Laboratory, Faculty of Bioscience, Universitat Autònoma de Barcelona, Barcelona, Spain; ^2^Department of Biology, Healthcare and Environment, Faculty of Pharmacy and Food Science, Universitat de Barcelona, Barcelona, Spain

**Keywords:** lead tolerance, cell wall, GWAS, extensins, TLC-domain

## Abstract

Soil contamination by lead (Pb) has become one of the major ecological threats to the environment. Understanding the mechanisms of Pb transport and deposition in plants is of great importance to achieve a global Pb reduction. We exposed a collection of 360 *Arabidopsis thaliana* natural accessions to a Pb-polluted soil. Germination rates, growth, and leaf Pb concentrations showed extensive variation among accessions. These phenotypic data were subjected to genome wide association studies (GWAs) and we found a significant association on chromosome 1 for low leaf Pb accumulation. Genes associated with significant SNP markers were evaluated and we selected EXTENSIN18 (EXT18) and TLC (TRAM-LAG1-CLN8) as candidates for having a role in Pb homeostasis. Six Pb-tolerant accessions, three of them exhibiting low leaf Pb content, and three of them with high leaf Pb content; two Pb-sensitive accessions; two knockout T-DNA lines of GWAs candidate genes (*ext18, tlc*); and Col-0 were screened under control and high-Pb conditions. The relative expression of *EXT18*, *TLC*, and other genes described for being involved in Pb tolerance was also evaluated. Analysis of Darwinian fitness, root and leaf ionome, and TEM images revealed that Pb-tolerant accessions employ two opposing strategies: (1) low translocation of Pb and its accumulation into root cell walls and vacuoles, or (2) high translocation of Pb and its efflux to inactive organelles or intracellular spaces. Plants using the first strategy exhibited higher expression of *EXT18* and *HMA3*, thicker root cell walls and Pb vacuolar sequestration, suggesting that these genes may contribute to the deposition of Pb in the roots. On the other hand, plants translocating high amounts of Pb showed upregulation of *TLC* and *ABC* transporters, indicating that these plants were able to properly efflux Pb in the aerial tissues. We conclude that *EXT18* and *TLC* upregulation enhances Pb tolerance promoting its sequestration: *EXT18* favors the thickening of the cell walls improving Pb accumulation in roots and decreasing its toxicity, while *TLC* facilitates the formation of dictyosome vesicles and the Pb encapsulation in leaves. These findings are relevant for the design of phytoremediation strategies and environment restoration.

## Introduction

Lead is a neurotoxic element that even at low concentrations is highly dangerous for humans and animals. Due to its former use as gasoline additive, this contaminant is distributed all over the world with urban and heavy-traffic roadside areas mostly affected (Levin-Schwartz et al., 2021). The primary sources of Pb in urban soils are weathering paint, transportation, irrigation, soil amendments and industrial waste/debris (Clark et al., 2006; Ratul et al., 2018). In the case of agricultural soils, Pb release is generally attributed to metalliferous mining, smelting activities and the use of fertilizers and pesticides (Reboredo et al., 2019; Yuan et al., 2019). Plants, in contrast to humans, are relatively tolerant to soil Pb. Toxicity symptoms in the form of chlorosis, leaf browning, and stunted growth usually occur only in soils with close to 1000 mg/kg. Nonetheless, phytotoxicity depends on the metal speciation and physicochemical properties of soil such as pH, cation exchange capacity, and the presence of ligands notably affect the metal speciation and bioavailability (Smith, 2009). In fact, a Pb^2+^ activity as low as 0.06 μM was found to be highly toxic to roots of cowpea (Kopittke et al., 2007).

Pb enters the plant mainly by soil–root transfer. However, foliar uptake is an important second pathway in areas with high fallout of airborne particles (Uzu et al., 2010; Luo et al., 2019). Plants growing in Pb-contaminated soils accumulate the metal mainly in the roots. Fortunately, only a small proportion is translocated to consumable tissues (Yousaf et al., 2016). Consumption of plants cultivated on Pb-rich soils is the greatest potential health risk above exposure to soil/dust or drinking tap water (Byers et al., 2020). Therefore, it is important to grow crops with low ability for Pb accumulation in edible parts.

Although non-hyperaccumulator species usually restrict Pb transport to the shoots, leaf Pb accumulation may considerably vary among species (Bech et al., 2012; Ng et al., 2016; Zhou et al., 2016) and even varieties (Liu et al., 2013). The mechanisms behind such differential Pb accumulation in plants are still poorly understood. One of the principal mechanisms to prevent the toxic effect of metals in plants is the absorption on cell wall components (Gupta et al., 2013). Pb^2+^ binds tightly to carboxyl groups in cell walls and pectin has been proposed as a main ligand for lead (Inoue et al., 2013; Chudzik et al., 2018). Methyl-esterification of cell wall pectins reduces availability of carboxyl groups for metal immobilization. In fact, a substantial increase of low methyl-esterified pectins has been observed in Pb-induced cell wall thickenings in different plant species (Krzesłowska et al., 2010; Sumranwanich et al., 2018).

Environmental signals produce modifications in the composition and chemical structure of cell walls. Related to metal stress, the thickening of xylem and cortical parenchyma cell walls has been reported as a phytotoxic effect of metal exposition in *Brachiaria decumbens* (Gomes et al., 2011). After polysaccharides (cellulose, hemicelluloses, and pectins), proteins are the second major components of cell walls having structural and enzymatic functions. Although proteins represent around 10% of cell wall, their activities are responsible for the cell wall alterations during plant development and environmental stress (Cruz-Valderrama et al., 2019). The first described cell wall proteins were the Extensins (EXTs), insoluble glycoproteins that strengthen the primary cell wall and also participate in defense (Lamport et al., 2011). Furthermore, EXTs can interact with other cell wall components such as pectins (Qi et al., 1995). Many extensin genes have been cloned and their expression can be regulated and induced by biotic and abiotic stressors (Josè-Estanyol and Puigdomènech, 2000; Wei and Shirsat, 2006). Different studies have reported extensin induction after wounding, however, to date they have not been associated with Pb stress. On the contrary, soluble glycosylated proteins of cell wall (AGPs, arabinogalactan proteins) have been related to metal toxicity showing negative expression due to metal exposure (Mareri et al., 2019).

Molecular genetic approaches to identify Pb transport mechanisms are still not conclusive. It has been argued that Pb, as a non-essential element, may not be moved in plants by specific transporters. In fact, HMA3, a metal transporter of the P-type ATPase family, seems to be involved in vacuolar transport not only of Pb, but also of Cd, Co, and Zn (Morel et al., 2009). Additional transporter genes involved in lead tolerance have been identified in Arabidopsis: the ABC genes *ATM3*, *PDR8*, and *PDR12*, and the *ACBP1* (Acyl-CoA-binding domain protein) (Lee et al., 2005; Kim et al., 2006, 2007; Xiao et al., 2008). Moreover, under Pb stress *Arabidopsis thaliana* induces the expression of the cytoplasmatic protein PSE1 (Fan et al., 2016). The authors reported a certain connexion of this gene with the transporter *PDR12* because *PSE1* activates genes involved on the phytochelatin synthesis which could induce the expression of ABC transporters.

Recently developed genetic tools are being applied to discover new molecular markers associated to lead tolerance. One of them is the genome-wide association study (GWAs), used to connect plant phenotypes with candidate marker genes. This tool has lately been used to describe nine potential candidate genes related to the Pb-tolerance in *Brassica napus* (Zhang et al., 2020). Using the GWAs approach, we aim to improve the fragmentary picture of genes responsible for Pb tolerance taking advantage of the variability present in natural accessions of *A. thaliana*. In particular, our goals are (1) to elucidate the mechanisms developed by Pb-tolerant accessions that differ in their Pb sequestration strategies, and (2) to stablish the role of the novel candidate genes identified by GWAs in Pb tolerance.

## Materials and Methods

### *HapMap* Phenotyping and GIS Data Extrapolation

Seeds of the *Arabidopsis thaliana* HapMap cohort (360 natural populations) were obtained from Nottingham *A. thaliana* Stock Centre (NASC, Nottingham, United Kingdom). The list and information of the accessions is detailed in Supplementary Dataset 1. Seeds were surface sterilized using 3:10 bleach solution in constant agitation for 10 min and rinsed with sterile water six times. Seeds were stratified for 4 days at 4°C in 0.1% agar solution to synchronize germination, then five seeds per accession were sown in two soil types with contrasted Pb concentrations into pot trays. The control soil was a mix of commercial potting mix soil with perlite (2:1); the mine soil was a mix of soil from an ancient lead mine (Mina de Can Vergeli; 41°53′42.4′′N 3°01′11.7′′E), commercial potting mix soil and perlite (2:1:1). Trays were placed in a growth chamber with 150 mmol/m^2^s of light intensity, 12 h light/12 h dark photoperiod, and 25°C/20°C day/night temperature. Trays were bottom-watered twice weekly with deionized water. Germination and survival were monitored and rosette diameter (RD) of three individuals per accession and soil type was measured weekly for 4 weeks. Growth rate of each accession was calculated as mean(RD_Mine_)_/_mean(RD_Control_) of the last RD measure. Fifty-day old plants were harvested to measure its nutrient mineral contents.

In order to estimate the edaphic parameters of each *A. thaliana* natural population native soil, coordinate locations and public maps from the European Soil Data Centre (ESDAC) database (Panagos et al., 2012) were combined using Q-GIS^1^. Natural populations coordinates were extracted from GWAPP^2^ in WGS84 system (latitude and longitude). Maps of heavy metals in the soils of the EU, based on Lucas, 2009 HM data, were used to estimate the Pb, As, Cd, Cr, Cu, Hg, Mn, Sb, Co, and Ni concentrations (mg/kg) of the European topsoils. Maps of soil properties at European scale, based on Lucas, 2009/2012 topsoil data, were used to extract the following variables: pH (measured in H_2_O), pH (in CaCl_2_ 0.01 M solution), Cation Exchange Capacity (CEC), Calcium carbonates (CaCO_3_), C:N ratio, and N, P, and K concentrations (mg/kg). Native soil data was available for 305 of the 360 *A. thaliana HapMap* cohort.

### GWA Studies and Candidate Genes Selection

Phenotypes obtained from the 210 *A. thaliana* natural accessions that survived and grew on the mine soil were used for Genome Wide Association Studies (GWAs). These phenotypes included: (1) Rosette Diameter mean (RD_Mine_) of three 50-days old plants cultivated in the mine soil, (2) growth rate [RD_Mine/Control_ = mean(RD_Mine_)_/_mean(RD_Control_)] of three 50-day old plants, (3) native soil Pb concentration, and (4) leaf mineral concentrations (Pb, Zn, Cu, B, Ca, K, Mg, Na, P, S, Fe, Mn) of three plants per accession cultivated in the mine soil (Supplementary Dataset 1). All the phenotypes are detailed in Supplementary Dataset 1.

The *HapMap* population has been genotyped for 250K bi−allelic SNPs (Platt et al., 2010; Chao et al., 2012), and this dataset is available in the GWA−Portal platform^3^ (Seren, 2018). GWAs were performed under the following settings: the accelerated mixed model, which considers the population structure; 250 k SNP dataset; and BoxCox transformation (normality was checked by applying the Shapiro–Wilk test). After running the analysis, the filtering of the minor allele frequency (MAF) was set to 0.1. SNPs above Bonferroni correction [–log10(*p*) > 5] were selected, and the linkage disequilibrium (LD) region associated with the highest score SNP was explored to determine the candidate genes in LD. GWAs output for each phenotype are available in Supplementary Dataset 2.

To validate the significant SNPs obtained on the GWA-Portal, genotype data of the 210 *A. thaliana* accessions was obtained from 1001genomes.org/(1135_snp-short-indel.vcf.gz). Indels were removed and SNPs were filtered using the criteria allele count < 100 and MAF < 4.8% using GATK-3.6.0 (McKenna et al., 2010). Principle components analysis (PCA) was performed and a kinship matrix was calculated using the GAPIT package in R (Lipka et al., 2012). The compressed mixed linear model (CMLM) was used for performing GWAs by incorporating K matrix along with PCAs employing the program TASSEL 5.0 (Bradbury et al., 2007; Supplementary Table 1).

All genes associated with the significant SNPs and their SNPs in strong LD (*r*^2^ > 0.8) were explored (Supplementary Table 1). Genes were annotated according to TAIR 10 (Araport11) and relative gene expression data for *A. thaliana* (Col−0) plants exposed to lead was retrieved from a previous transcriptome study (unpublished data).

### Plant Materials and Experimental Design

**FIGURE 1:**
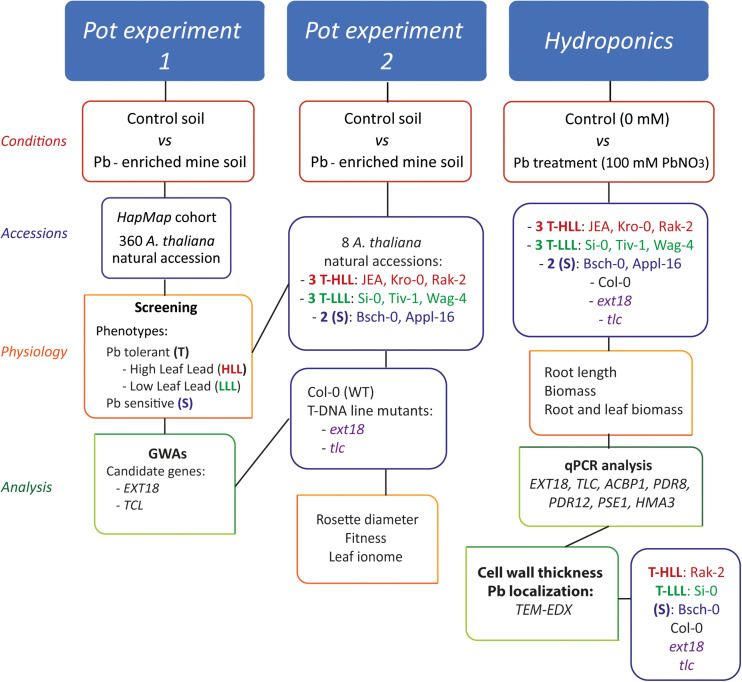
Methodology process flowchart.

Seeds from eight selected *A. thaliana* natural populations (Si-0, Tiv-1, Wag-4, JEA, Kro-0, Rak-2, Appl-16, and Bsch-0), Col-0 and 2 T-DNA insertion lines (*ext18* (SALK_201747C), and *tlc* (SALK_136500) mutants) were obtained from the *A. thaliana* Stock Centre (NASC, Nottingham, United Kingdom). Figure 1 details the workflow and the *A. thaliana* accessions and mutants used in each experiment. *A. thaliana* natural accessions were classified as “High Leaf Lead” (HLL) or “Low Leaf Lead” (LLL) based on their leaf Pb concentrations, and as tolerant (T) or sensitive (S) accessions based on their growth under high-Pb conditions.

### Pb-Enriched Mine Soil Pot Experiment

Seeds were stratified for 4 days at 4°C in 0.1% agar solution to synchronize germination, then sown into the same soil types (Mine/Control) used for the *HapMap* screening. Each tray contained 11 accessions (Si-0, Tiv-1, Wag-4, JEA, Kro-0, Rak-2, Appl-16, Bsch-0, Col-0, *ext18* and *tlc)* randomized, with a total of eight individuals per accessions and soil type. Plants were allowed to germinate and grow at 12 h light/12 h dark and 25 to 20°C day/night temperature for 1 week before transferring them to a 4°C environment at 8h day length for 6 weeks of vernalization to synchronize bolting time between the accessions. After the vernalization period, trays were moved back to the growth room at initial conditions. Trays were bottom-watered twice weekly with 250 ml of deionized water per tray. Rosette diameter was measured every week for 5 weeks and the number of siliques was counted at maturity. For leaf ionome analysis, two leaves from 5-week-old plants were collected from four plants per accession and soil type.

### Hydroponic Experiment

Seeds of the 11 accessions (Si-0, Tiv-1, Wag-4, JEA, Kro-0, Rak-2, Appl-16, Bsch-0, Col-0, *ext18*, and *tlc)* were surface sterilized with bleach solution (30% commercial bleach + 0.02% Triton X-100) for 15 min and washed five times with sterile water and sown in plates with: half MS medium (pH 4.5, Control) or half MS medium and 50 μM of PbNO_3_ (pH 4.5, Pb treatment). Plates were kept in the dark at 4°C for 48 h to synchronize germination. After that, plates were placed in a growth chamber with 150 mmol/m^2^s of light intensity, 12 h light/12 h dark photoperiod, and 25°C/20°C day/night temperature. Radicle emergence and root length were monitored for 15 days. Six 10-day old seedlings per accession and treatment were transferred to individually hydroponic circular containers (50 mL) filled with 0.5-strength Hoagland solution (pH 4.5) and 0 μM (control) or 50 μM of PbNO_3_. The hydroponic solution was changed every 3 days to maintain a constant concentration of nutrients in the solution and PbNO_3_ was increased three times until a final concentration of 100 μM of PbNO_3_ (pH 4.5, Pb treatment) when plants were 21 days old. Plants remained at these conditions for 2 weeks. We harvested 35-days old plants and we measured the root length, rosette diameter and total fresh weight (biomass) of six individuals per accession and treatment. Roots and leaves from four plants per accessions were stored for ionomic and microscopy analysis.

### Elemental Composition of Soils and Plants

To analyze the composition of the control and mine soils, we took six independent samples of each soil type from the trays used for the pot experiment, three at the begging, and three at the end of the experiment. The soil characterization was performed on the 2-mm fraction samples following the extraction method described in Busoms et al. (2018).

Plant tissues were sampled by removing 2–3 leaves or cutting the roots (1–5 mg dry weight) and washed it with 18 MΩ water before being placed in Pyrex digestion tubes. Sampled plant material was dried for 2 days at 60°C and weighed before open-air digestion in Pyrex tubes using 0.7 mL concentrated HNO3 at 110°C for 5 h in a hot-block digestion system (SC154-54-Well Hot Block, Environmental Express, Charleston, SC, United States). Concentrations of the selected elements (Ca, K, Mg, Na, P, S, Mo, Cu, Fe, Mn, Zn, Pb, and Cd) were determined by ICP-MS (Perkin ElmerInc., ELAN 6000, MA, United States) or ICP-OES (Thermo Jarrell-Ash, model 61E Polyscan, England).

### Gene Expression Analyses

Total RNA of about 100 mg of plant powder was extracted using the Maxwell^®^ RSC plant RNA kit (Promega Corporation, WI, United States) following the manufacturer’s instructions. Two micrograms of total RNA were used as a template to synthesize first-strand cDNA with the iScript^TM^ cDNA Synthesis Kit (Bio-Rad, United States). The cDNA was used as a template for quantitative PCRs using iTaq^TM^ Universal SYBR^®^ Green Supermix (Bio-Rad, CA, United States). Real-time detection of fluorescence emission was performed on a CFX384 Real-Time System (Bio-Rad, CA, United States), and plates were edited using the CFX manager version 3.1 software.

Primers used for *EXT18* (At1g26250), *TLC* (At1g26200), *ACBP1* (At5g53470), *PDR8* (At1g59870), *PDR12* (At1g15520), *PSE1* (At5g06370), and *HMA3* (At4g30120) transcript quantification are detailed in Supplementary Table 2. For normalization across samples, the expression of the *ACTIN 2* gene (At3g18780) was analyzed. For each sample, the average value from triplicate real-time PCRs was used to estimate transcript abundance. The mean Ct values were normalized against *Actin2* gene and Ct values were calculated as (Ct*_Gene_*- Ct*_*Actin*__1_*). The relative expression of each target gene was calculated with the 2^–ΔΔ*Ct*^ method (Pb treatment – control) in four samples per accession and treatment (Livak and Schmittgen, 2001).

### TEM Imaging

To determine ultrastructural changes and location of Pb deposition, plant material from four individuals of Si-0, Rak-2, Bsch-0, Col-0, *ext18*, and *tlc* accessions submitted to control (0 μM PbNO_3_) and high Pb conditions (100 μM PbNO_3_) in hydroponics for 2 weeks was harvested. Roots and leaves were fixed using 3% (v/v) glutaraldehyde in 0.1M cacodylate buffer overnight at 4°C. After washing three times in buffer, plant material was post fixed with 1% (v/v) osmium tetroxide in the medium buffer for 1 h and washed twice in distilled water. Samples were dehydrated in a graded acetone series followed by infiltration and embedded in Spurr’s resin. Roots and leaves were sectioned using a glass knife on Leica EM UC7 ultramicrotome (Leica Microsystems, Wetzlar, Germany). Roots were continuously oriented transversely to the root axis to allow recording of the distance from the root tip by counting sections. Ultrathin (80 nm) sections were made using a Diatome diamond knife on Leica EM UC7, collected onto copper grids, then treated with 5% uranyl acetate in water for 60 min. Sections were examined in a Jeol 1200 TEM (Jeol, Tokyo, Japan). Cell wall thickness was measured using ImageJ software. At least 30 cells per accession, organ and treatment were measured. For Pb identification, sections were examined using a TEM-EDX spectroscopy system JEOL-1400 (Jeol, Tokyo, Japan).

### Statistical Analyses

All the statistical analyses were conducted using JMP SAS software (SAS Institute, Cary, NC, United States). Normality was checked and non-normal data were transformed before applying any parametrical tests. Mean-standardized values (–1 < value > 1) of elemental contents of soil and leaf material were used to represent the radar plots and compare between soil types, treatments or accessions. Outlier removal was implemented using the algorithm described in Davies and Gather (1993).

The phenotypic responses to high Pb conditions were quantified and compared to the respective control conditions [relative measurements: mean(*X*_Pb_)_/_mean(*X*_Control_)]. We used a *linear mixed effects model (LMM)* to investigate changes in rosette diameter (RD) and silique number (Fitness) in the pot experiment, and changes in root length (RL) and fresh weight (Biomass) in the hydroponic experiment. The models included a two-way interaction between ‘Pb treatment’ and ‘accession’ or ‘accession classification.’

One-way ANOVA was used to test for significant differences (*P* < 0.05) between means of phenotypic responses, gene expression, and between means of elemental contents of soil and leaf material. To test for correlations between two variables a Bivariate Fit was conducted. To perform multiple comparisons of group means we used Tukey’s HSD. Statistical data analyses are specified in Supplementary Datasets 3, 4.

### Data Availability

The data generated and/or analyzed in the current study are either included in this article as Supplementary Material or submitted to public repositories. Raw phenotype data are available from AraPheno^4^ and full GWAs analysis are available at Gwa-portal under the study named ‘Leaf Pb Mine^5^’.

## Results

### Screening of *Arabidopsis thaliana HapMap* Tolerance to Pb Stress

To investigate the Pb tolerance of natural accessions of *A. thaliana*, the 360 accessions constituting the *HapMap* cohort (Horton et al., 2012) were cultivated in a Pb-enriched soil excavated from an old, abandoned metal mine in the NE of Spain (Mine “Can Vergeli”; Supplementary Figure 2A). In parallel, the same cohort was cultivated in a control non-metalliferous soil with similar characteristics (Supplementary Figure 1). Of the 360 accessions sown, 296 germinated and grew well under control conditions; 226 germinated in both soil types and only 210 accessions survived more than 5 weeks in the mine soil (Supplementary Dataset 1). Although the rosette diameter (RD) of 5 weeks old plants varied significantly among all the accessions under both control and Pb stress conditions, the RD of almost all the accessions was reduced under high Pb conditions (RD_Mine/Control_ mean ± SD: 0.67 ± 0.18). However, we detected a group of accessions with great tolerance to Pb stress (RD_Mine/Control_ > 0.9, Supplementary Dataset 1).

**FIGURE 2:**
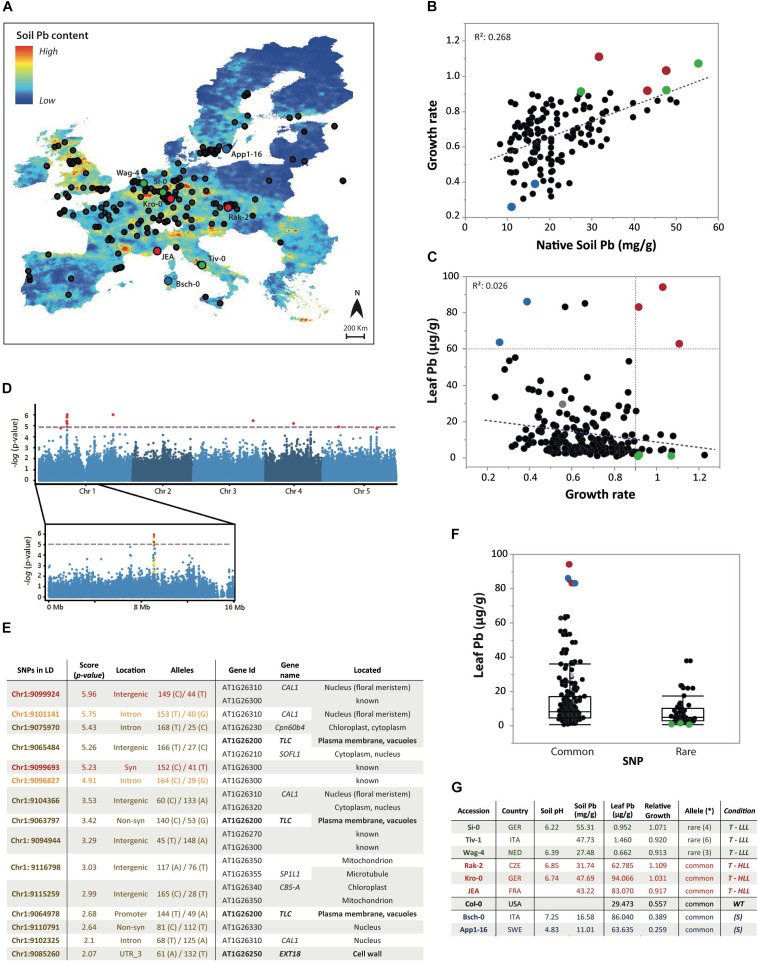
GWAS of *A. thaliana* HapMap leaf Pb content. **(A)** Europe map of estimated soil Pb content with the geographic location of the European *A. thaliana* populations from the HapMap cohort (black dots). Selected accessions are highlighted with colored circles and names. **(B)** Estimated Pb concentration of the native soil of 141 European *A. thaliana* accessions and the relationship with the growth rate (Rosette diameter of 42-day-old plants) of each accession cultivated in common soil (control) or in Pb-enriched mine soil (mine). **(C)** Leaf Pb concentration of 210 accession cultivated in Pb-enriched mine soil for 6 weeks and the relationship with the growth rate (RD_Mine/Control_). We considered Pb-tolerant accessions (T) when growth rate is >0.9, and Pb sensitive accessions (S) when growth rate is <0.4. **(D)** Manhattan plot of all *A. thaliana* chromosomes and zoom of chromosome 1 region with a significant peak for SNP associations to leaf Pb content. The horizontal purple dashed line corresponds to a nominal 0.05 significance threshold after Bonferroni correction. SNPs are color-coded to show their LD relationships with the top SNP (yellow = 0.4 *r*^2^ < 0.6; orange = 0.6 *r*^2^ < 0.8; red = *r*^2^ > 0.8). **(E)** Position, score (*p*-value), location, and allele description of the 15 SNPs in high LD; target genes of each SNP and its cellular localization. **(F)** Leaf Pb concentration of the two haplotypes identified: “low leaf lead” (LLL) associated with the rare allele, and “high leaf lead” (HLL) associated with the common allele. **(G)** Description of the nine selected *A. thaliana* natural accessions. (*) Indicates the number of SNPs in which they have the rare allele. Selected accessions are highlighted with colored dots in all the graphs: green dots = T-LLL; red dots = T-HLL; blue dots = (S) and gray dot = Col-0.

To inspect if this tolerance was associated with soil properties from the native habitats of the tested accessions, we used the public maps from the European Soil Data Centre (ESDAC) to extrapolate the pH and trace metal content of all the European sites (Figure 2A). We observed that plants coming from sites with elevated soil Pb content (and acidic pH) had better capacity to tolerate this stress (Figure 2B). This confirms the importance of soil composition as a potential factor driving local adaptation of plants.

Leaf tissue of three plants grown under both conditions was harvested for ionomic analysis. All the elements presented high variability depending on the accession and growth conditions. However, we detected no significant correlation between the Pb tolerance of the accessions (growth rate) and the concentration of any of the elements analyzed (*R*^2^ < 0.1) (Supplementary Dataset 1). The most relevant differences were found in leaf Pb accumulation from plants grown in the mine soil (RSD = 1.2). We observed two clear phenotypes among the most tolerant accessions: those accumulating high amounts of Pb in the shoots, denoting a good Pb compartmentalization mechanism; and those not translocating the Pb to the aerial parts, suggesting Pb exclusion or root accumulation strategies (Figure 2C). We classified the *A. thaliana* natural accessions as ‘High Leaf Lead’ (HLL) or ‘Low Leaf Lead’ (LLL) based on their leaf Pb concentrations, and as tolerant (T) or sensitive (S) accessions based on their growth rate (RD_Mine/Control_).

### Detection of SNPs Associated With Low Pb Content in Above-Ground Tissues

To identify genomic regions underlying tolerance to Pb, we conducted GWAs on several phenotypes including growth (RD_Mine_ and RD_Mine/Control_) of 50-day old plants, native soil Pb content, and leaf mineral content of plants cultivated in the mine soil (see section “Materials and Methods”). The only trait that gave significant associations was the leaf Pb content of natural accessions exposed to a Pb-enriched mine soil (Supplementary Dataset 2). Significantly associated SNPs [-log10(*p*) > 5] were identified in chromosomes 1 (7 SNPs), 3 (1 SNP), 4 (1 SNP), and 5 (2 SNPs) (Figure 2D). The scores, positions, genes associated with these SNPs, and the LD of the regions are detailed in Supplementary Table 1. Here, we focused on the most significant peak of chromosome 1 but we consider that the peaks found in Chr4 and Chr5 should be investigated in future studies.

We explored the 15 SNPs in strong LD (*r*^2^ > 0.8) with the top SNP of Chr1, obtaining a total of 12 candidate genes (Figures 2D,E). For all the SNPs, the rare allele was associated with the ‘Low Leaf Lead’ (LLL) haplotype (Figures 2F,G). The function, localization, and expression of the candidate genes was examined. We selected *EXT18* (At1g26250) and *TLC* (At1g26200) because we considered they were the only target genes that could have a role in Pb sequestration. EXT18 is a member of the 20 ‘classical EXTs’ considered structural components of primary cell walls (Johnson et al., 2003). Its localization in the cell walls could contribute the deposition of Pb in these cell structures. TLC is a protein with a TRAM/LAG1/CLN8 lipid-sensing domain involved in several lipid-related processes. Its role in Golgi transport vesicle could enhance Pb encapsulation. To verify if these genes were responsible for the observed LLL association we obtained the knockout mutants of these genes (*ext18, tlc*) to conduct further studies.

### Phenotypic Responses to High Pb

**FIGURE 3:**
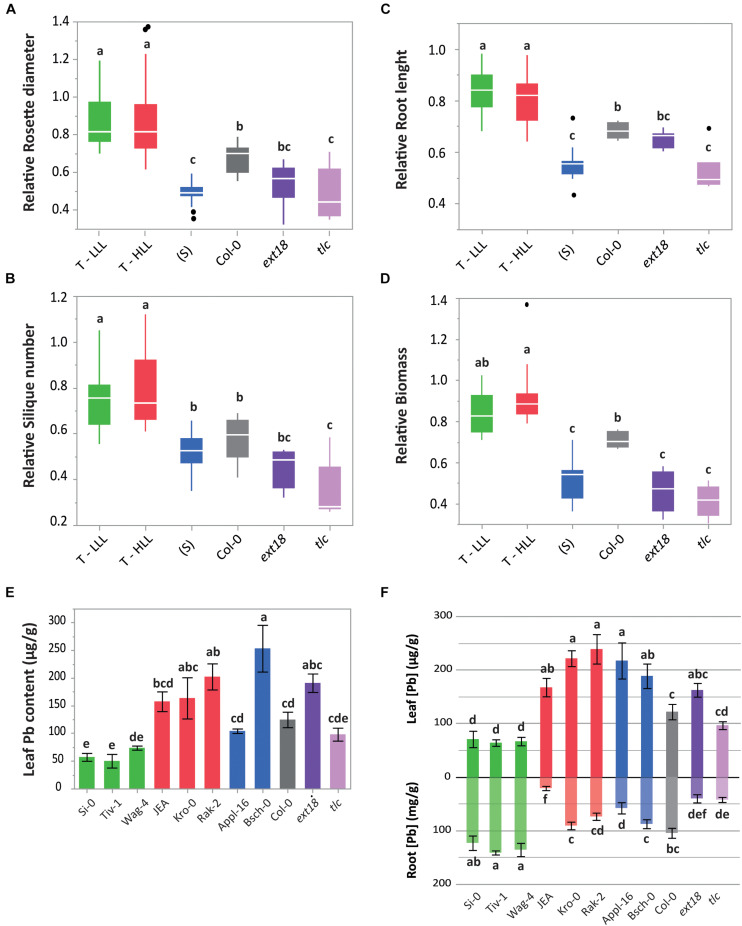
Phenotyping of representative accessions under Pb stress. Mean ± SE of **(A)** growth rate (RD_Mine/Control_) and **(B)** fitness (silique number) of 3 T-LLL (green), 3 T-HLL (red), 2 (S) (blue) accessions, Col-0 (gray), *ext18* (dark purple), and *tlc* (light purple) cultivated in a pot experiment in control soil or in Pb-enriched mine soil (*n* = 8 plants per accession and soil type). Mean ± SE of **(C)** root length (mm) and **(D)** biomass (fresh weight, g) of 3 T-LLL, 3 T-HLL, 2 (S) accessions, Col-0, *ext18*, and *tlc* cultivated in hydroponic solution and treated with 0 or 100 μM of PbNO_3_ for 2 weeks (*n* = 6 plants per accession and treatment). Mean ± SE of **(E)** leaf Pb content from four plants per accession cultivated in the Pb-enriched mine soil; **(F)** leaf and root Pb content of four plants per accession cultivated in hydroponics and treated with 100 μM of PbNO_3_ for 2 weeks. Letters indicate significant differences (*p* < 0.05, Tukey’s HSD).

To confirm the tolerance or sensitivity of the 11 selected accessions, we repeated the same pot experiment performed to conduct the GWAs but with a higher number of replicates (*n* = 8 individuals per accession and soil type). In parallel, in order to conduct root analysis, the same accessions were cultivated in hydroponics and treated with 0 μM (Control) or 100 μM of PbNO_3_ (Pb treatment). The phenotypic responses to high Pb conditions were quantified and compared to the respective control treatments. Both experimental settings verified that accessions classified as tolerant (T), independently of being LLL or HLL, had larger rosettes, longer roots and produced more seeds than the other accessions when they were cultivated under Pb stress (Figures 3A–D). Col-0 again showed an intermediate tolerance and both mutants, *tlc* and *ext18*, performed similar to the sensitive accessions, suggesting that the missing genes have a role in Pb tolerance. We noticed that all the tested accessions suffered a root length reduction under the Pb treatment, but *ext18* also had shorter roots under control conditions (Supplementary Figure 2).

The analysis of ionome denoted that our hydroponic experiment mimicked the mine soil conditions because in both settings we obtained similar patterns in the leaf nutrient composition of the different accessions (Supplementary Figure 3). As observed by other authors, Pb reduces nutrient concentration in shoots, especially of bivalent cationic elements such as Zn, Mn, Mg, Ca (Pourrut et al., 2011). In our pot experiment we did not detected a decrease of tissue Zn levels, probably because the mine soil used was also rich in Zn, as well as in other trace elements like Cd and Cu (Supplementary Figure 1). However, in the hydroponic experiment we could clearly observe a Pb-induced reduction of most of the divalent nutrients in the shoots (Supplementary Figure 3).

Leaf ionome analysis corroborated the LLL and HLL condition of the selected accessions (Figures 3E,F). We also detected that *ext18* mutants translocated high levels of Pb, hinting that this mutant is not able to retain the Pb in the cell wall of the roots (Figures 3E,F). Importantly, root ionome analysis revealed that LLL accessions accumulate high amounts of Pb in the roots (Figure 3F), thus they cannot be considered Pb excluders and must have exceptional mechanisms of root Pb deposition. On the other hand, both T-HLL and Pb-sensitive (S) accessions translocated similar Pb concentrations (Figure 3F), suggesting that (S) plants are not able to maintain the Pb out of the cytosol and it becomes toxic for them. The *tlc* mutant, despite accumulating less Pb in the leaves than the HLL accessions, exhibited similar growth and fitness performance than the (S) plants (Figure 3), indicating the costs of Pb toxicity.

### Transcript Levels of Genes Involved in Pb Tolerance

**FIGURE 4:**
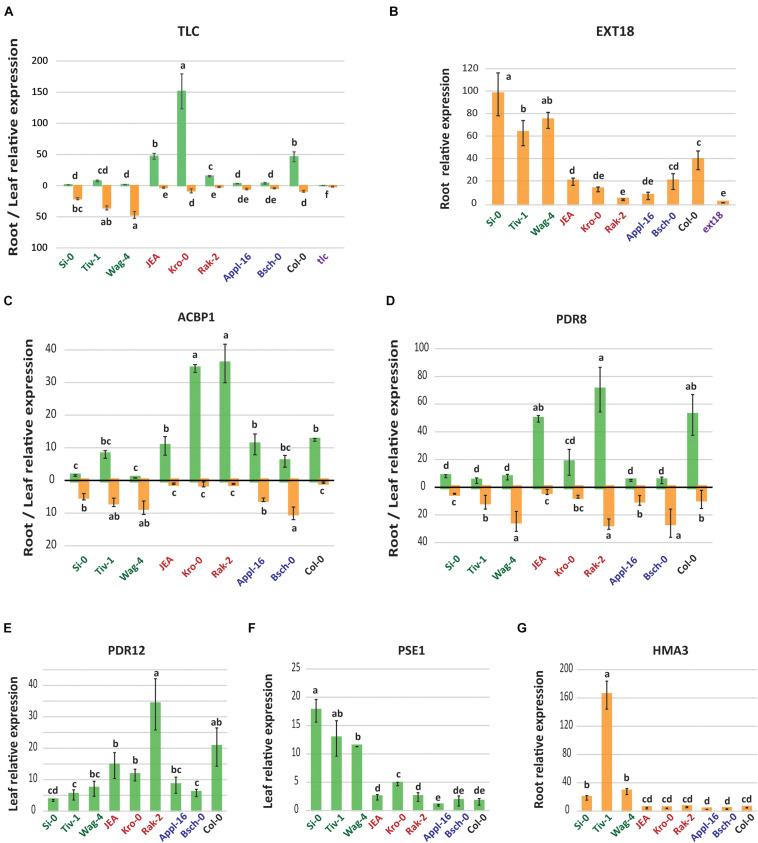
Transcript expression of GWAs target genes and other genes involved in Pb tolerance. Leaf and root relative expression (2^– ΔΔ*Ct*^) of **(A)** *TLC* (At1g26200), **(C)** *ACBP1* (At5g53470), and **(D)** *PDR8* (At1g59870). Root relative expression (2^– ΔΔ*Ct*^) of **(B)** *EXT18* (At1g26250) and **(G)** *HMA3* (At4g30120). Leaf relative expression (2^– ΔΔ*Ct*^) of **(E)** *PDR12* (At1g15520) and **(F)** *PSE1* (At5g06370). Data represents the mean ± SE of four plants per accession and letters indicate significant differences (*p* < 0.05, Tukey’s HSD).

Gene-expression followed by proteins synthesis is also affected by Pb stress. We compared the expression level of *TLC* and *EXT18* in plants exposed to 0 or 100 μM of PbNO_3_ for 2 weeks. We detected that the *TLC* gene was induced by Pb in the leaves of T-HLL and Col-0 plants and in the roots of the T-LLL accessions (Figure 4A). The transcription of *TLC* correlated with the amount of Pb found in the tissues of the tolerant plants, suggesting again that this gene can enhance Pb tolerance.

*EXT18* only expresses in plants roots and we found that Pb treatment induced the expression of this gene in almost all the tested accessions (Figure 4B). However, *EXT18* expression in roots of T-LLL plants was much higher than in the other plants. *TLC* and *EXT18* expression of the respective mutants was undetectable in any tissue under any condition (Supplementary Dataset 4), confirming that the T-DNA insertions used to generate the *tlc* and *ext18* mutants are blocking its transcription (Figures 4A,B).

Several ABC (ATP-binding cassette) transporters, such as ACBP1, PDR8, or PDR12, have been identified as being involved in resistance to Pb (Kim et al., 2006, 2007; Xiao et al., 2008). In our tested accessions these three genes were highly upregulated in the leaves of T-HLL plants, especially in the Rak-2^*T–HLL*^ accession (Figures 4C–E). *ACBP1* and *PDR8* were enhanced by Pb in the roots of the T-LLL accession but also in the roots of the (S) plants (Figures 4C,D). Our results indicate that the regulation of these transporters is more relevant in aerial tissues, where they can be involved in Pb sequestration into inactive organelles.

*PSE1* is a gene described for conferring Pb tolerance and root Pb accumulation in *A. thaliana* (Fan et al., 2016). Our T-LLL accessions displayed much higher expression of *PSE1* than the rest of accessions (Figure 4F), supporting that this gene is induced under Pb stress and confers tolerance preventing Pb translocation.

Intracellular sequestration is a further relevant mechanism to minimize Pb toxicity to vital functions of the cytosol. The HMA3 protein is involved in Pb detoxification by participating in its vacuolar sequestration (Morel et al., 2009). We detected that Tiv-1^*T–LLL*^ and the other T-LLL accessions also manifested higher levels of *HMA3* expression in their roots (Figure 4G).

### Cell Wall Thickening and Pb Deposition

**FIGURE 5:**
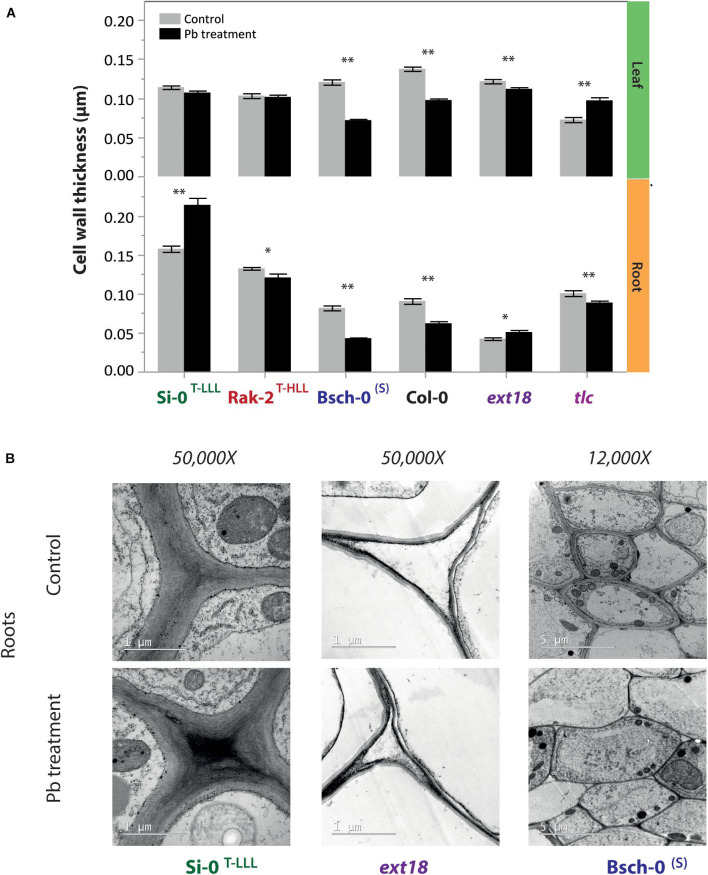
Cell wall thickness of representative accessions under control and high Pb conditions. **(A)** Wall thickness of leaf cell (mesophyll) and root cells (cortex) of Si-0, Rak-2, Bsch-0, Col-0, *ext18*, and *tlc* plants cultivated in hydroponic solution and treated with 0 μM (control, gray bars) or 100 μM of PbNO_3_ (Pb treatment, black bars) for 2 weeks. Data represents the mean ± SE of 30 cells from three plants per accession. Significant differences between treatments are marked with asterisks (*p < 0.05, **p < 0.001; Student’s *t*-test). **(B)** Transmission electron microscopy (TEM) images of root cortex cells of Si-0, ext18 (50,000 magnification) and Bsch-0 (12,000 magnification) plants submitted to control or Pb treatment and showing the differences in wall thickness between accessions and treatment.

Cell wall thickening and lignification are important histological responses of the plants to avoid metal toxicity. To verify whether the presence of Pb or the upregulation of *EXT18* stimulates the thickness of the cell walls, we measured the roots and leaves cells walls of Si-0^*T–LLL*^, Rak-2^*T–HLL*^, Bsch-0^*S*^, Col-0, *ext18*, and *tlc* plants exposed to control or 100 μM PbNO_3_ for 2 weeks. The major differences were identified in root cells walls. Pb stress caused a pronounced thinning of the root cell walls of the sensitive accessions Bsch-0^*S*^ and *ext18* (Figure 5). On the contrary, Si-0^*T–LLL*^ individuals exhibited wider root cell walls when plants were treated with Pb (Figure 5).

**FIGURE 6:**
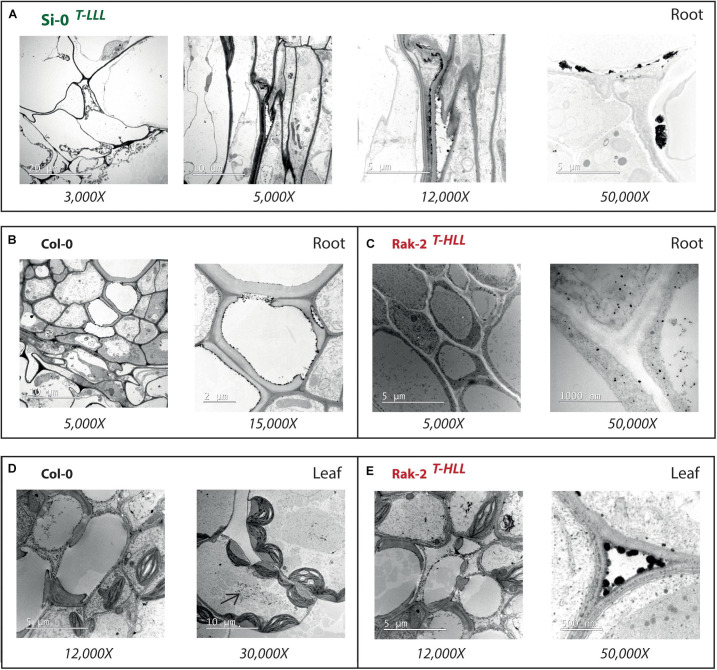
Lead localization in *A. thaliana* root and leaf cells by TEM-EDX. **(A)** Cross sections of tolerant Si-0^*LLL*^ accession illustrating large Pb deposits saturating the root cell walls. Cross sections of root cortex cells from **(B)** Col-0 and **(C)** Rak-2^*HLL*^ accessions showing Pb deposits not precipitated into cell walls. Cross section of leaf mesophyll cells from **(D)** Col-0 and **(E)** Rak-2^*HLL*^ accessions exposing Pb deposits sequestered in vacuoles and intracellular spaces.

To determine the subcellular location of Pb, we performed TEM-EDX analysis in the same plants used for cell wall thickening measurements (Supplementary Figure 4). We observed large crystalline-like deposits that saturated the root cell walls of the tolerant Si-0^*T–LLL*^ accession (Figure 6A). TEM images also exposed that Rak-2^*T–HLL*^ plants had few Pb deposits in the roots (Figure 6C) but in the leaves they were able to efflux almost all the translocated Pb to intercellular spaces (Figure 6E). In comparison, we observed similar amount of Pb deposits in both roots and leaves of Col-0 plants (Figures 6B,D). These depositions were not clearly immobilized in root cell walls or intercellular spaces, but we detected a high sequestration in the leaf vacuoles (Figure 6D).

## Discussion

Lead is a non-essential metal that does not have a role in any cell metabolism process but it is easily absorbed and accumulated in various plant tissues. Excessive Pb accumulation impairs morphological, physiological and biochemical functions and has a range of deleterious effects, such as inhibition of root growth, chlorosis or impaired uptake of essential elements (Pourrut et al., 2013). Given this, metalliferous habitats exert clear selection pressures on plant communities (e.g., Sailer et al., 2018). We observed that the *HapMap* cohort of *A. thaliana* natural accessions come from habitats with remarkable differences in soil Pb content. Our screen of the 360 *A. thaliana* accessions in a Pb-enriched mine soil revealed a variety of responses to Pb stress, from accessions that did not germinate, to some that survived a few days, to accessions that grew even better in high Pb conditions than control soil. Among the tolerant accessions, we identified plants accumulating high levels of leaf Pb (termed HLL accessions) and plants with low leaf Pb (LLL accessions). We observed that accessions coming from sites with acidic soils and elevated levels of Pb were the most tolerant to this stress, manifesting signals of local adaptation.

Numerous studies have documented local adaptation of plants to environmental factors and soil characteristics (e.g., Antonovics, 2006; Busoms et al., 2015), but fewer have focused on adaptations to specific soil pollutants. An important condition for natural selection to act upon a trait is that this trait has phenotypic variation which is genetically determined. Pb uptake, translocation and accumulation in plants can be regulated by several genes that are still unknown. GWAs has become a standard procedure for dissecting complex genetic traits, with proven success in plants (e.g., Fusari et al., 2017). In order to detect novel genes related to Pb tolerance, we performed a GWA study on phenotypes obtained from the cultivation of 360 *A. thaliana* natural accessions in control and Pb-enriched mine soils.

Leaf Pb content from plants cultivated in the Pb-enriched mine soil was the only phenotype that showed significant associations. Significant SNPs from various chromosomes were explored but we focus on the chromosome 1 region where all the rare SNPs are associated with a unique phenotype: accessions with low leaf Pb concentrations (LLL). Except for hyperaccumulator species, metal tolerant plants tend to restrict soil–root and root–shoot transfers. Ionomic analysis revealed that T-LLL accessions were capable of accumulating Pb in the roots, restricting the translocation but not the uptake.

Pb has strong affinity for cell wall, membrane constituents and detoxification systems (Meyers et al., 2009; Krzesłowska et al., 2016). The two target genes, *EXT18* and *TLC*, located in these structures were selected to study their role in Pb sequestration and tolerance.

### Role of *EXT18* in Pb Tolerance

Extensins are cell wall-located, basic, Hyp-rich structural glycoproteins with alternating hydrophilic and hydrophobic motifs (Lamport et al., 2011). Extensin deposition is considered important for the correct assembly and biophysical properties of primary cell walls, with consequences for growth, cell adhesion, plant resistance to pathogens, and ion depositions (Roberts and Shirsat, 2006; Pereira et al., 2011). Cannon et al. (2008) demonstrated that the loss of the structural glycoprotein network in *A. thaliana* is lethal and therefore primary cell wall proteins are essential. Indeed, Choudhary et al. (2015) revealed that *EXT18* is an important contributor to cell wall integrity in rapidly extending walls, and it is essential for the normal vegetative growth of Arabidopsis plants. Extensins have been mainly associated with cell wall structure and biotic stress resistance. However, our results suggest that extensins are also relevant for metal detoxification.

Hydroponic cultivation of Pb tolerant (T-LLL and T-HLL) and sensitive (S) natural accessions, the *ext18* mutant and the wild type (Col-0) under control and high Pb conditions (100 μM PbNO_3_) revealed that Pb treatment enhanced *EXT18* expression in root of T-LLL and Col-0 accessions. Overexpression of EXTs promotes root hair growth (Marzol et al., 2018) and increases cell wall biomass (Tan et al., 2014). T-LLL accessions had much longer roots and extended root cell walls farther than the rest of accessions. Thicker cell walls are essential to accommodate Pb deposits. TEM images showed that root cell walls of T-LLL plants exposed to high Pb were covered with Pb deposits, preventing the translocating of Pb to the aerial tissues. Moreover, we observed that *ext18* mutants had thinner root cell walls than wild type (Col-0) plants and the mutants accumulated higher Pb levels in the leaves when submitted to Pb stress, supporting the hypothesis that without this gene plants have a reduced capacity to retain metals in their roots.

**FIGURE 7:**
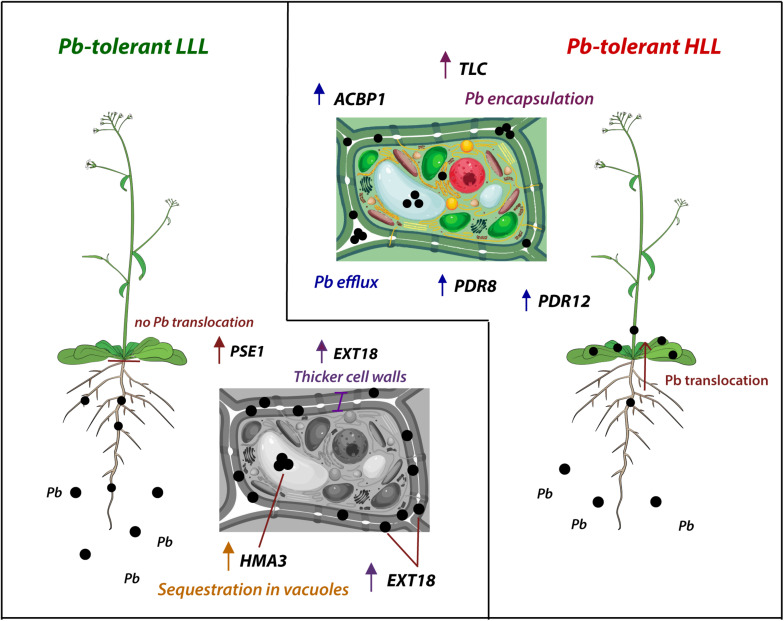
Model for the Pb tolerance mechanism of tolerant LLL (Low Leaf Lead) and HLL (High Leaf Lead) *A. thaliana* natural accessions.

Early studies revealed that oxygen peroxide causes enhanced transcription of extensin genes in tomato roots (Wisniewski et al., 1999). Peroxidation of cell wall EXTs is a fundamental process for cell wall strengthening, and EXT18 is able to form iso-di-tyrosin bonds thus providing covalent cross-linking networks that play a fundamental role in cell wall integrity (Choudhary et al., 2015). We thus speculate that the enhanced expression of *EXT18* found in the T-LLL accessions is contributing to the expansion of the root cell walls and the consequent Pb deposition (Figure 7).

### Potential Role of TLC-Domain in Pb Sequestration

TRAM/LAG1/CLN8 lipid-sensing domain has been well described in humans and yeast, where it is associated with ER-Golgi trafficking (Winter and Ponting, 2002). In plants, TLC is cataloged as a ‘lipid-sensing domain containing protein’ and its molecular function is unknown (Araport11). However, TLC was identified in a GWAs as a candidate gene with known relationships to lipid metabolism and protection against peroxidation in maize (Riedelsheimer et al., 2013).

Early studies by Malone et al. (1974) and Wierzbicka et al. (2007) reported Pb sequestration in Golgi apparatus, endoplasmatic reticulum, and plasma membrane. Results on Pb compartmentation in the Pb-resistant signal grass (*Brachiaria decumbens*) suggest that Pb locates to intracellular membrane structures, probably Golgi apparatus, prior to final deposition in cell walls (Kopittke et al., 2008). The endomembrane system is a complex network of membrane-bound compartments. The post-Golgi transport pathways are responsible for the deposition of many of the structural components of cell walls in the apoplast (Sinclair et al., 2018). Lead deposits in Golgi-derived vesicles has been interpreted as a way to efflux Pb to the apoplast by exocytosis (Malone et al., 1974).

*TLC* was upregulated in the leaves of T-HLL and Col-0 accessions and in the roots of T-LLL accessions submitted to a Pb treatment, in accordance with the Pb accumulation pattern of each phenotype. TEM images revealed that Col-0 and Rak-2^*T–HLL*^ plants retained Pb in leaf vacuoles, inactive organelles, or intracellular spaces. The strong expression of *TLC* under Pb exposure in T-HLL plants suggests that this gene has an important role in Pb compartmentalization and Pb efflux to the apoplast (Figure 7).

### Contribution of Other Genes Involved in Pb Tolerance

Most of the genes that have been identified in plants under Pb exposure are related to antioxidative enzymes, metal transporters and Pb chelating compounds (Kumar and Prasad, 2018). Our GWAs results were not confined into these categories. However, it is well known that metal tolerance in plants can be highly polygenic and therefore the enhancement of other genes may also contribute to the Pb tolerance observed in *A. thaliana* natural accessions. Pb entering the symplast is removed by the activity of efflux pumps present in the plasma membrane. These transporters are constitutive, but their gene expression is stimulated by Pb (Pourrut et al., 2013). The relative gene expression of three plasma membrane ABC transporters (*ACBP1*, *PDR8*, and *PDR12*) were assessed and we found that Pb exposure induced the transcription of these transporters especially in leaves of T-HLL plants. As noted by Jiang et al. (2017), these transporters may favor the active efflux of Pb followed by sequestration into inactive organelles or its transport to the cell exterior.

*PSE1* encodes an unknown protein but was described for promoting Pb tolerance in *A. thaliana* plants by Fan et al. (2016). In this study, the authors showed that *pse1-1* loss-of-function mutant was sensitive to Pb and the expression of *ABC* transporters was increased in *PSE1*-overexpressing plants subjected to Pb stress. Our expression analysis indicated that this gene was highly upregulated in the T-LLL accessions, supporting a role for it in Pb tolerance. Finally, we also evaluated the root expression of the vacuolar transporter HMA3. Morel et al. (2009) suggested that *HMA3* plays a role in Pb detoxification by participating in their vacuolar sequestration. Pb exposure significantly enhanced the expression of *HMA3* in our T-LLL accessions. It is possible that T-LLL plants take advantage of both strategies and the excess of Pb that is not deposited in the cell walls gets trapped in the vacuole.

In summary, *A. thaliana* has evolved two main strategies to cope with high substrate Pb: (1) exclusion from the shoot by sequestration in the roots and (2) translocation to the shoots and storage in inactive organelles or intracellular spaces. The first strategy involves upregulation of *EXT18* and *HMA3* in the roots favoring the thickening of the root cell walls and Pb vacuolar accumulation, improving Pb sequestration in roots and decreasing its toxicity (Figure 7). On the contrary, in plants with strategy 2, the higher expression of ABC transporters may enhance the Pb translocation to the aerial tissues. However, the upregulation of these transporters along with the *TLC* gene promotes efficient leaf Pb efflux to the apoplast and Pb encapsulation (Figure 7). We believe these two strategies are not exclusive to *A. thaliana* and extensins and similar lipid-domains should be explored in other plant species.

We conclude that the phenotypic and genomic variability present in worldwide *A. thaliana* natural accessions is a valuable resource to study molecular mechanism involved in Pb stress. Future studies with deeper sampling will surely discoverer additional genes associated with the sequestration of Pb and other environmental pollutants. Besides, given the success with *A. thaliana*, our findings suggest a bright future for developing native plants to be used for phytoremediation strategies.

## Data Availability Statement

The original contributions presented in the study are included in the article/Supplementary Material or in the public repositories indicated in the Materials and Methods section. Further inquiries can be directed to the corresponding author/s.

## Author Contributions

SB, ML, CP, and SM conceived the study. ML performed the GWA study. LP-M and SB conducted the laboratory experiments and data analyses. LP-M and SM performed the image analyses. SB wrote the manuscript with primary input from all authors. All authors edited and approved the final manuscript.

## Conflict of Interest

The authors declare that the research was conducted in the absence of any commercial or financial relationships that could be construed as a potential conflict of interest.

## Publisher’s Note

All claims expressed in this article are solely those of the authors and do not necessarily represent those of their affiliated organizations, or those of the publisher, the editors and the reviewers. Any product that may be evaluated in this article, or claim that may be made by its manufacturer, is not guaranteed or endorsed by the publisher.

## Footnotes

1
http://qgis.osgeo.org

2
http://gwapp.gmi.oeaw.ac.at/

3
http://gwas.gmi.oeaw.ac.at

4
https://arapheno.1001genomes.org/study/92/

5
https://arapheno.1001genomes.org/rest/study/12/values.csv

## Supplementary Material

Supplementary Figure 1 Properties of the experimental soils. **(A)** Map of Catalonia indicating the location of the Pb/Zn mine where soil was excavated. **(B)** Radial plot of normalized difference of 13 elements from control and mine soils. **(C)** Elemental composition and the pH of the control and mine soils. Data represents the mean ± SE of six samples per soil type. Elements exhibiting significant differences are marked with an asterisk (^∗^).

Supplementary Figure 2 Growth and fitness of each *A. thaliana* accession cultivated under Pb stress conditions. Mean ± SE of **(A)** rosette diameter (cm) and **(B)** fitness (silique number) of each accession cultivated in a pot experiment in control soil (gray) or in Pb-enriched mine soil (orange) (*n* = 8 plants per accession and soil type). Mean ± SE of **(C)** root length and **(D)** biomass (fresh weight, g) of each accession cultivated in hydroponic solution and treated with 0 μM (gray) or 100 μM of PbNO_3_ (orange) for 2 weeks (*n* = 6 plants per accession and treatment).

Supplementary Figure 3 Ionomic profiles of each *A. thaliana* Pb-phenotype and *ext18* and *tlc* mutants exposed to control and high Pb conditions. **(A)** Radial plots of normalized differences of 12 elements from leaves of 3 T-LLL, 3 T-HLL, 2 (S) accessions and Col-0 cultivated in a pot experiment in control soil (gray) or in Pb-enriched mine soil (orange). Radial plots of normalized differences of 12 elements from **(B)** leaf ionome and **(C)** roots of 3 T-LLL, 3 T-HLL, 2 (S) accession and Col-0 cultivated in hydroponic solution and treated with 0 μM (gray) or 100 μM of PbNO_3_ (orange) for 2 weeks.

Supplementary Figure 4 TEM-EDX analysis of Pb deposits. **(A)** Example images of analyzed spectrums. **(B)** EDX weight and atomic ratios of spectrums focused in distinct areas.

Supplementary Table 1 Significant SNPs information and associated genes. The score of each SNP was obtain from the GWA-Portal (AMM analysis) and from Tassel software (CMLM analysis).

Supplementary Table 2 Primers list for transcript quantification of target genes.

Supplementary Dataset 1 HapMap cohort information and phenotypes analyzed of the 360 accessions cultivated in a Pb-enriched mine soil.

Supplementary Dataset 2 SNPs positions and scores of all the phenotypes used for GWAs.

Supplementary Dataset 3 Statistical analysis of the pot and hydroponic experiments.

Supplementary Dataset 4 Statistical analysis of gene transcript analyses and cell wall measurements.
