# Supplementary material for: Genome-Wide Association Study Reveals Key Genes for Differential Lead Accumulation and Tolerance in Natural Arabidopsis thaliana Accessions
Source: Front Plant Sci. 2021 Aug 6;12:689316. doi: 10.3389/fpls.2021.689316 (PMC8377763; doi:10.3389/fpls.2021.689316)
Supplement: Supplementary Figure 1 — Properties of the experimental soils. (A) Map of Catalonia indicating the location of the Pb/Zn mine where soil was excavated. (B) Radial plot of normalized difference of 13 elements from control and mine soils. (C) Elemental composition and the pH of the control and mine soils. Data represents the mean ± SE of six samples per soil type. Elements exhibiting significant differences are marked with an asterisk (∗). [file Image_1.PDF]

(A)

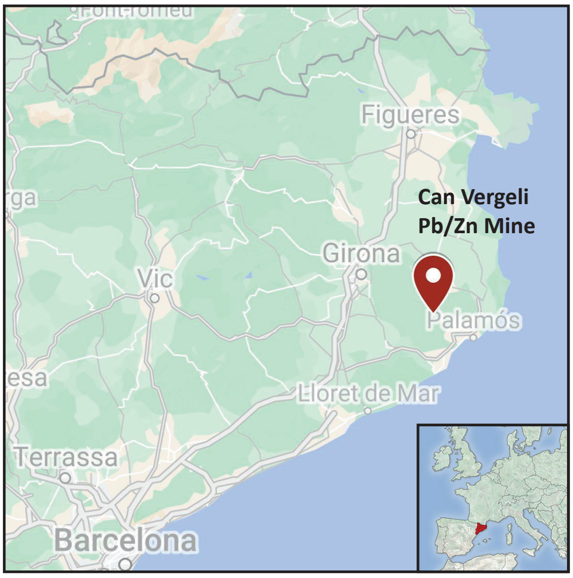

(B)

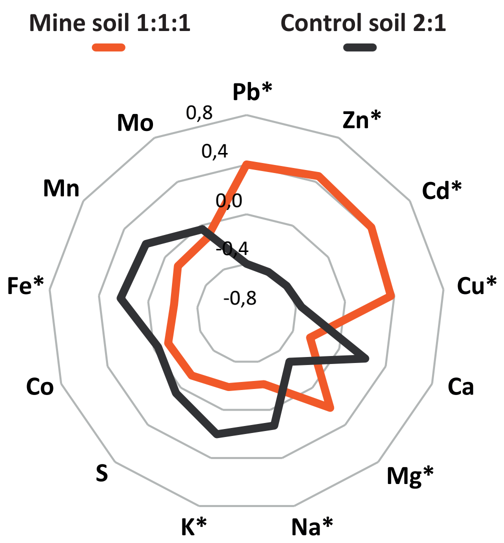

(C)

Element (µg/g)	*Control Soil Mean ± SE*	*Mine Soil Mean ± SE*
**Pb**	2.44 ± 23.02	419.27 ± 23.02
**Zn**	3.39 ± 2.20	51.71 ± 2.20
**Cu**	3.32 ± 0.54	11.87 ± 0.54
**Cd**	0.18 ± 0.04	1.08 ± 0.04
**K**	275.87 ± 6.38	195.68 ± 6.38
**Mg**	72.43 ± 1.62	90.04 ± 1.62
**Na**	59.58 ± 2.98	46.20 ± 2.98
**Fe**	27.94 ± 1.07	23.58 ± 1.07
**Mn**	176.14 ± 11.04	154.65 ± 11.04
**Co**	1.04 ± 0.04	1.01 ± 0.04
**Ca**	236.78 ± 17.68	181.91 ± 17.68
**Mo**	0.02 ± 0.01	0.02 ± 0.01
**S**	22.16 ± 2.07	18.47 ± 2.07
**pH**	6.8 ± 0.3	5.3 ± 0.4
