# Supplementary figures and images for: Genome-Wide Association Study Reveals Key Genes for Differential Lead Accumulation and Tolerance in Natural Arabidopsis thaliana Accessions
Source: Front Plant Sci. 2021 Aug 6;12:689316. doi: 10.3389/fpls.2021.689316 (PMC8377763; doi:10.3389/fpls.2021.689316)

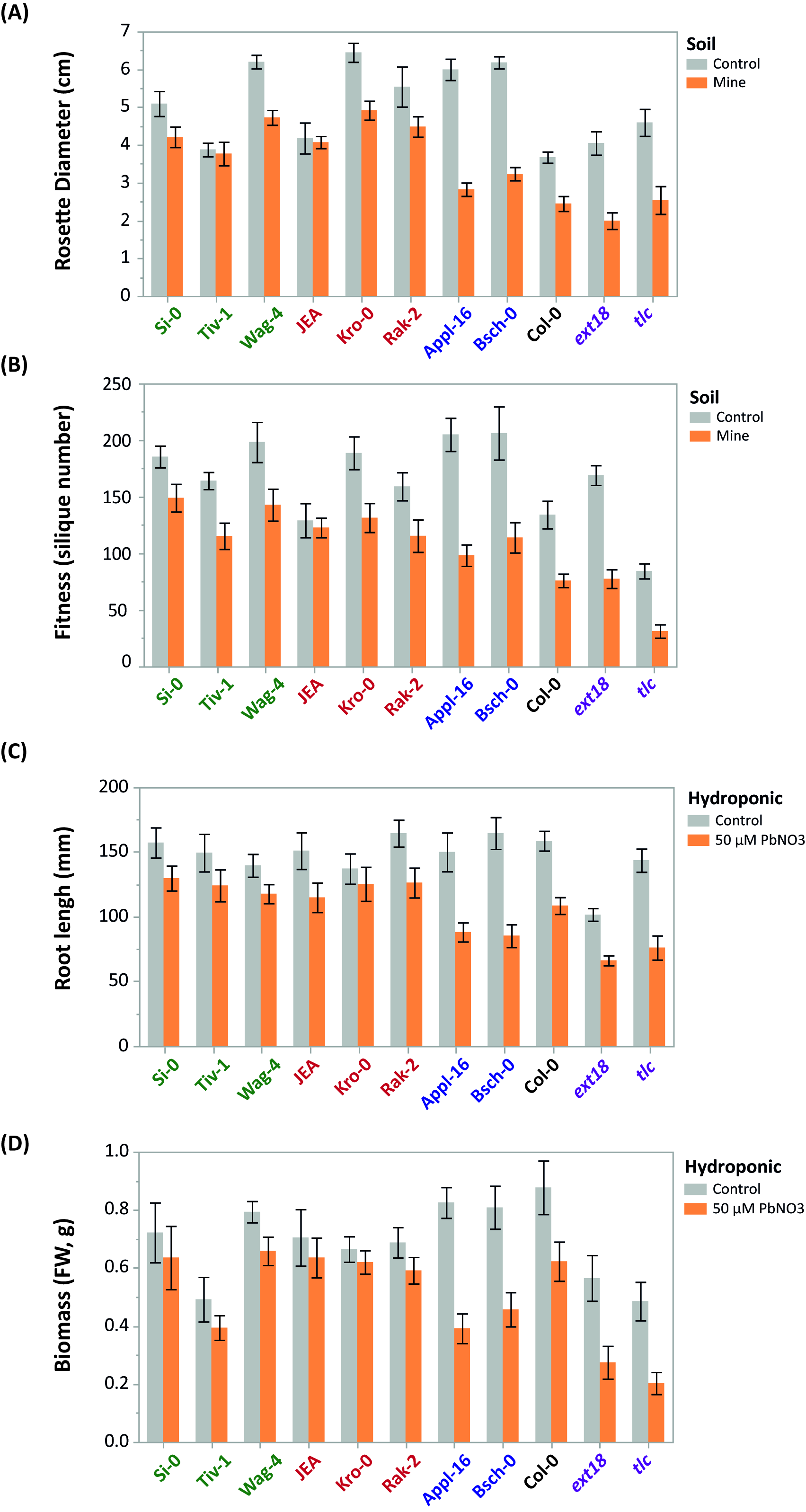

Supplement: Supplementary Figure 2 — Growth and fitness of each A. thaliana accession cultivated under Pb stress conditions. Mean ± SE of (A) rosette diameter (cm) and (B) fitness (silique number) of each accession cultivated in a pot experiment in control soil (gray) or in Pb-enriched mine soil (orange) (n = 8 plants per accession and soil type). Mean ± SE of (C) root length and (D) biomass (fresh weight, g) of each accession cultivated in hydroponic solution and treated with 0 μM (gray) or 100 μM of PbNO3 (orange) for 2 weeks (n = 6 plants per accession and treatment). [file Image_2.TIF]

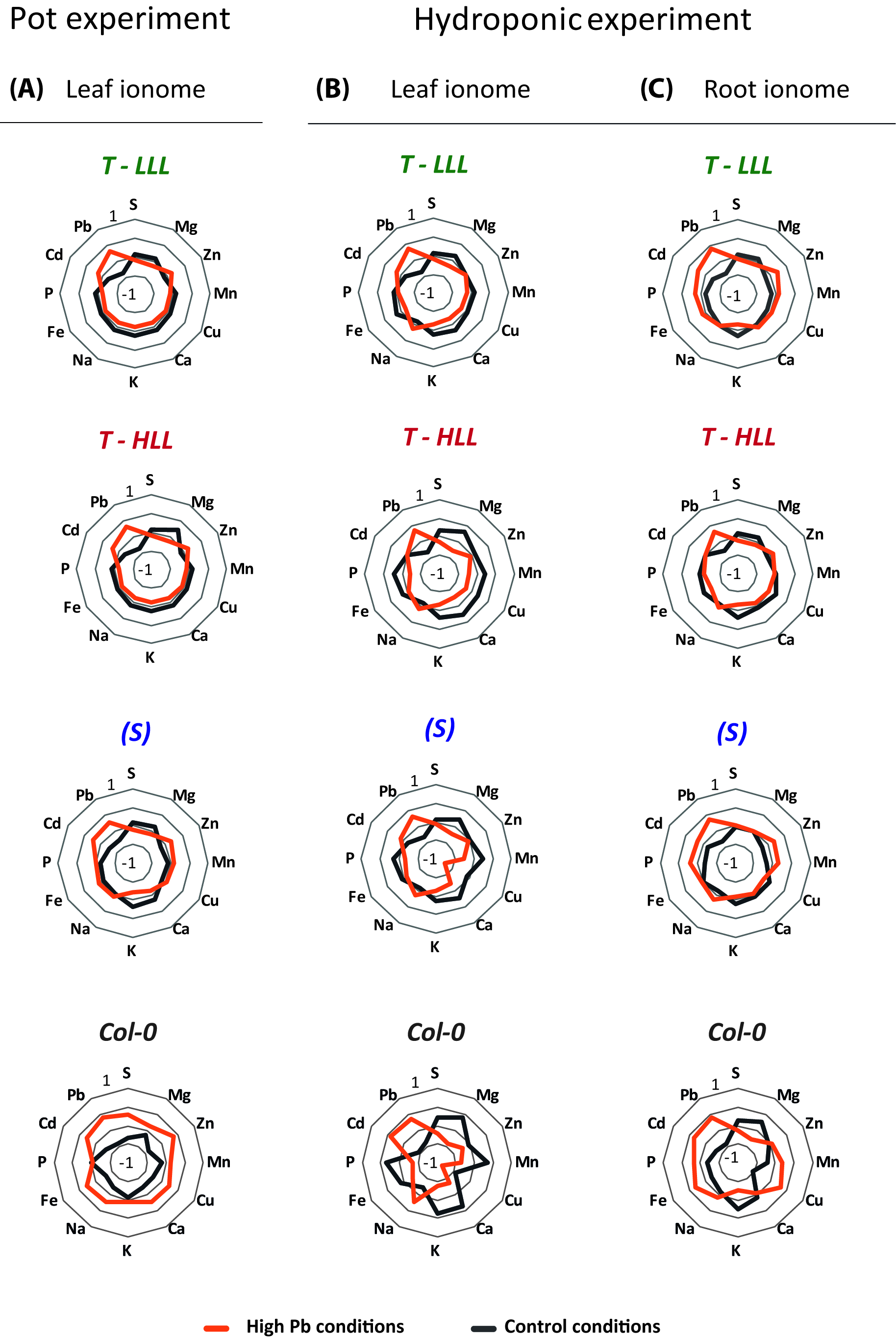

Supplement: Supplementary Figure 3 — Ionomic profiles of each A. thaliana Pb-phenotype and ext18 and tlc mutants exposed to control and high Pb conditions. (A) Radial plots of normalized differences of 12 elements from leaves of 3 T-LLL, 3 T-HLL, 2 (S) accessions and Col-0 cultivated in a pot experiment in control soil (gray) or in Pb-enriched mine soil (orange). Radial plots of normalized differences of 12 elements from (B) leaf ionome and (C) roots of 3 T-LLL, 3 T-HLL, 2 (S) accession and Col-0 cultivated in hydroponic solution and treated with 0 μM (gray) or 100 μM of PbNO3 (orange) for 2 weeks. [file Image_3.TIF]

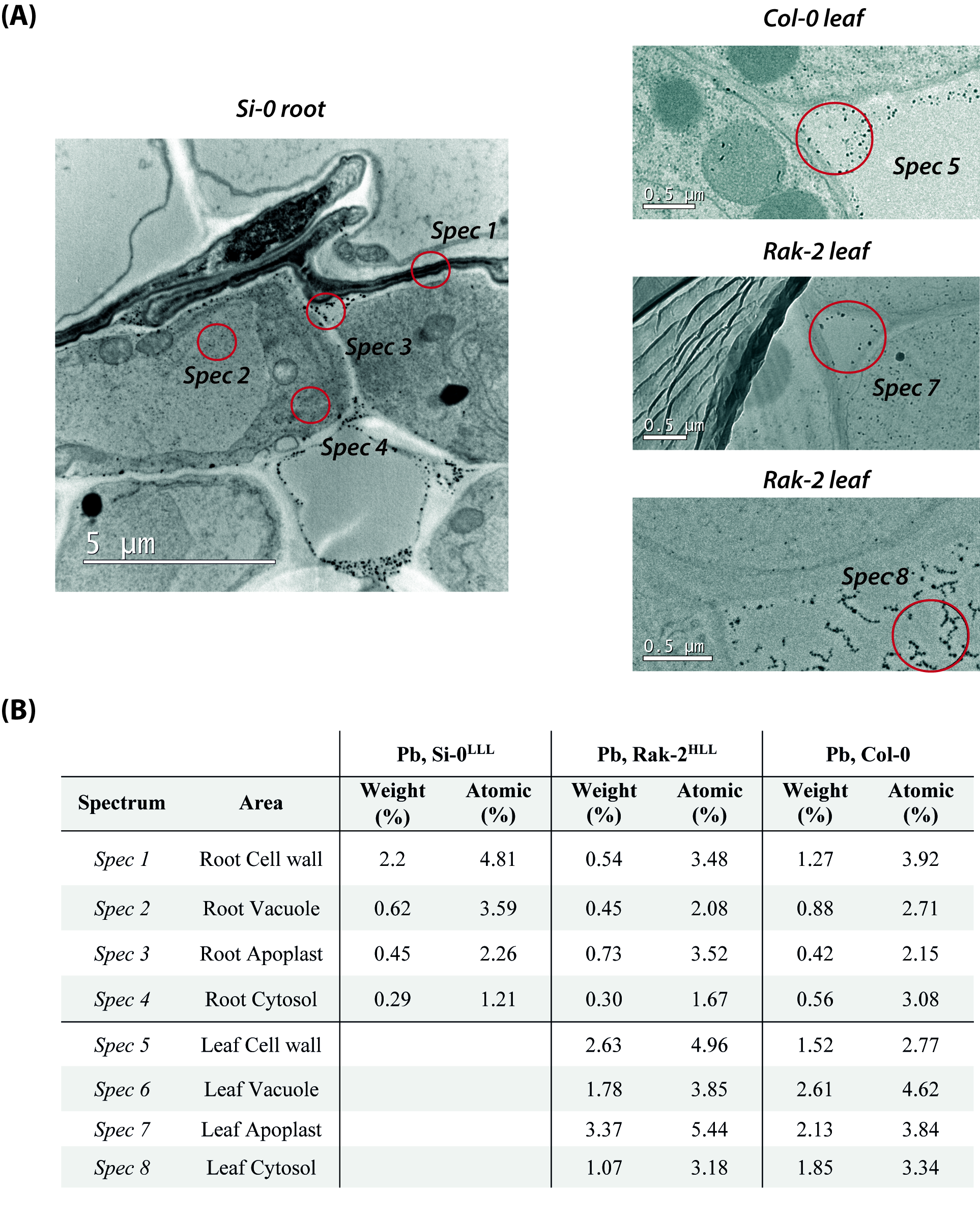

Supplement: Supplementary Figure 4 — TEM-EDX analysis of Pb deposits. (A) Example images of analyzed spectrums. (B) EDX weight and atomic ratios of spectrums focused in distinct areas. [file Image_4.TIF]
