# Supplementary material for: Genome-Wide Association Study Reveals Key Genes for Differential Lead Accumulation and Tolerance in Natural Arabidopsis thaliana Accessions
Source: Front Plant Sci. 2021 Aug 6;12:689316. doi: 10.3389/fpls.2021.689316 (PMC8377763; doi:10.3389/fpls.2021.689316)
Supplement: Supplementary Table 1 — Significant SNPs information and associated genes. The score of each SNP was obtain from the GWA-Portal (AMM analysis) and from Tassel software (CMLM analysis). [file Table_1.docx]

**Supplementary Table 1**: Significant SNPs information and associated genes. The score of each SNP was obtain from the GWA-Portal (AMM analysis) and from Tassel software (CMLM analysis).

**
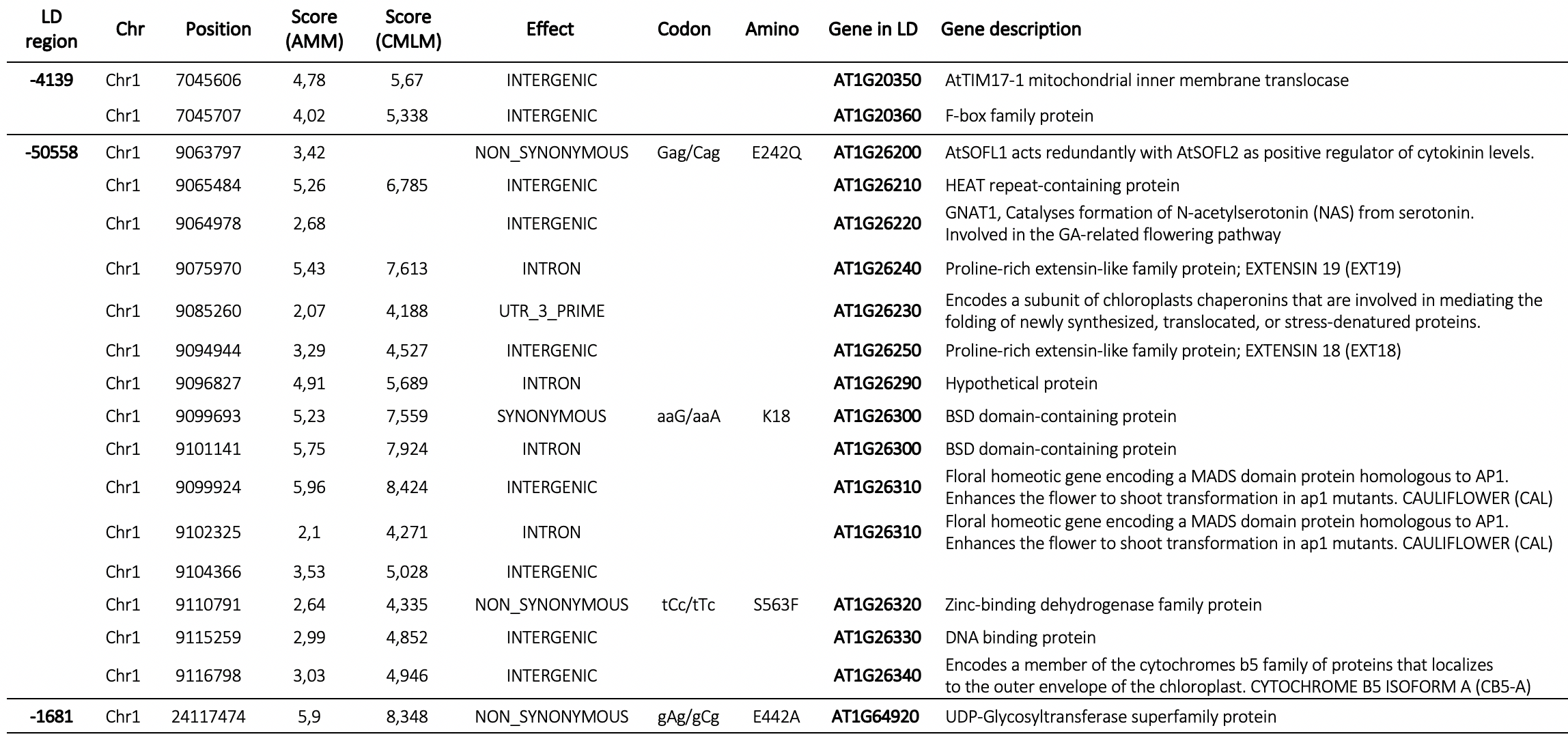
**

**
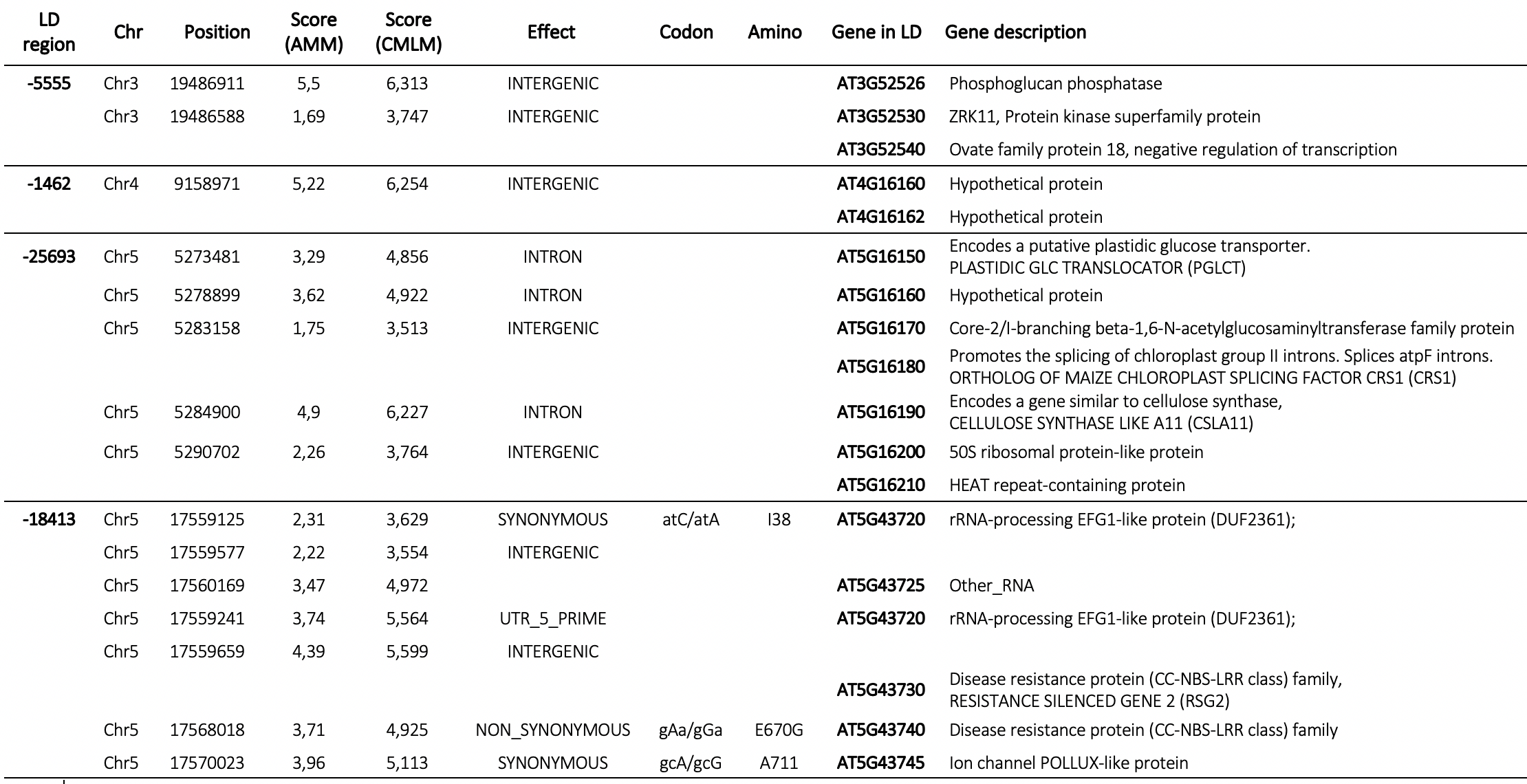
**
