# Supplementary material for: Genome-Wide Association Study Reveals Key Genes for Differential Lead Accumulation and Tolerance in Natural Arabidopsis thaliana Accessions
Source: Front Plant Sci. 2021 Aug 6;12:689316. doi: 10.3389/fpls.2021.689316 (PMC8377763; doi:10.3389/fpls.2021.689316)
Supplement: Supplementary Table 2 — Primers list for transcript quantification of target genes. [file Table_2.docx]

**Supplementary Table 2:** Primers list for transcript quantification of target genes.

| **Gene name** | **Primer** | **Product length** |
| --- | --- | --- |
| ACT2_F | TCGCTGACCGTATGAGCAAA | 151 |
| ACT2_R | TTGGAGATCCACATCTGCTG |  |
| EXT18_F | ATGTTTACAGCTCGCCACCGCC | 140 |
| EXT18_R | CCTCCACCACCTCCATACGTCGA |  |
| TLC_F | ACCTCCGCATCAGTGGTTTT | 141 |
| TLC_R | TGCAAAGTAGCCGCATGAGA |  |
| HMA3_F | TCTGCTGTTCATCGGAGGTT | 157 |
| HMA3_R | GCTTGATTCAGAGCCTTGACG |  |
| PDR8_F | GCCTGGGGAAATGAGAGACC | 148 |
| PDR8_R | TCCGGCCAAAACGTCCATAA |  |
| PDR12_F | GGACCTTGGAGGCAAAACGA | 203 |
| PDR12_R | TCTCGATGAAAACCTGAGCGA |  |
| ACBP1_F | ACTGCTCCTCAACCATCAGC | 178 |
| ACBP1_R | CAGCCTCACCACTCCGATTT |  |
| PSE1_F | CTCAGGTCCATCCCGAAACC | 165 |
| PSE1_R | CCTCCTCTGGCTTTGACGAG |  |
